# A novel heterozygous *MKRN3* nonsense mutation in a Chinese girl with idiopathic central precocious puberty: A case report

**DOI:** 10.1097/MD.0000000000022295

**Published:** 2020-09-18

**Authors:** Meijuan Liu, Lijun Fan, Chun Xiu Gong

**Affiliations:** Department of Endocrinology, Genetics and Metabolism, Beijing Children's Hospital, Capital Medical University, National Center for Children's Health, Beijing, China.

**Keywords:** case report, central precocious puberty (CPP), *MKRN3*, mutation

## Abstract

**Rationale:**

Central precocious puberty (CPP) is caused by the premature activation of the hypothalamic-pituitary-gonadal axis. Recently, the makorin ring finger protein 3 (MKRN3) mutations represent the most common genetic defects associated with CPP. However, the MKRN3 mutation is relatively rare in Asian countries. Here, we identified a novel heterozygous MKRN3 nonsense mutation (p. Gln363^∗^) causing CPP in a Chinese girl.

**Patient concerns:**

The index case is a 7-year-old Chinese girl who presented rapidly progressive precocious puberty with the onset of menstrual period 2 months after breast development, the advanced bone age (11 years), and the accelerated growth velocity (10 cm/year). Her basal luteinizing hormone (LH) and follicle-stimulating hormone (FSH) levels, as well as the peak LH/FSH values after the gonadotropin-releasing hormone (GnRH) stimulation test were significantly elevated.

Pelvic B ultrasound showed the presence of ovarian follicles with diameters ≥0.4 cm. Uterine length also indicated the onset of puberty. Contrast-enhanced magnetic resonance imaging (MRI) did not disclose any abnormality in the pituitary. Additionally, our present case was obese companies with impaired glucose tolerance (IGT) at the baseline assessment. Genetic analysis revealed a novel heterozygous nonsense mutation (c1087C>T; p. Gln363^∗^) in the maternally imprinted *MKRN3*, which inherited from the girl's father.

**Diagnosis:**

Combined with the symptoms, hormonal data, and the results of the pelvic B ultrasound, the girl was diagnosed as CPP.

**Interventions:**

The girl has been treated with a GnRH analog (3.75 mg every 4 wks) for 1 year and 5 months.

**Outcomes:**

The puberty signs have since not progressed during the follow-up period, which indicates that the GnRH analogs treatment is effective.

**Lessons:**

This case was obese companied with IGT at the baseline assessment and exhibited stronger LH/FSH response to GnRH stimulation test. Therefore, clinicians should highlight the importance of weight management and the long-term follow-up to monitor the adverse health outcomes, especially for the polycystic ovary syndrome in later life.

## 1 Introduction

Precocious puberty is clinically defined by the onset of secondary sexual characteristics before the age of 8 years in girls and 9 years in boys.^[1]^ Central precocious puberty (CPP), also well known as gonadotropin-dependent precocious puberty or true precocious puberty, is a condition caused by the early reactivation of the hypothalamic-pituitary-gonadal axis.^[2]^ CPP is characterized by the sequential breast development, pubic hair growth, and the menarche in girls, and of testicular enlargement, penile, and pubic hair growth in boys. The prevalence of CPP is sexually dimorphic, being predominantly in girls (girls: boys = 15–20:1).^[2]^ In contrast to CPP in boys, for whom 40% to 90% of precocious puberty have structural central nervous system abnormalities, 90% of girls with CPP are idiopathic.^[3]^ Recently, population-based studies have provided compelling evidence supporting the crucial roles of genetic effects on determining pubertal timing.^[4]^ Thus, the genetic analysis should be performed in girls with idiopathic CPP.

To date, a handful of genes, including kisspeptin 1 (*KISS1*) and its receptor (*KISS1R*), prokineticin receptor 2 (*PROKR2*), nuclear receptor subfamily 0 group B member 1 (*NR0B1*), delta-like non-canonical Notch ligand 1 (*DLK1*), and makorin ring finger protein 3 (*MKRN3*) have been demonstrated to be associated with CPP.^[5]^*MKRN3* is an intronless gene located on chromosome 15q11-q13 in the critical region associated with the Prader-Willi syndrome (PWS).^[6]^*MKRN3* is a maternally imprinted gene that the paternal allele is unmethylated, but the maternal allele is methylated.^[5]^ That is to say, only when the *MKRN3* mutations transmitted from the father that is associated with pathological phenotypes. In 2013, Abreu et al^[7]^ using whole-exome sequencing methods firstly identified *MKRN3* mutations as one of the causative factors of CPP in 5 of 15 families. Since then, more than 20 different loss-of-function genetic defects have been described, and *MKRN3* mutations have been identified as the most common genetic cause of CPP.^[8]^ Indeed, many experts suggest that *MKRN3* mutations should be suspected in all familial and idiopathic CPP cases and *MKRN3* gene analysis should be included in the routine clinical investigation of CPP.^[2,9]^

Herein, we report a new heterozygous *MKRN3* nonsense mutation (p. Gln363^∗^) causing CPP in a 7-year-old Chinese girl presented with the development of breast, the onset of menstrual period, the advanced bone age (11 yrs) and the accelerated growth velocity (10 cm/yr).

## 2 Case presentation

A Chinese girl at 7 years and 7 months was referred to our clinic due to the premature thelarche and menarche. The thelarche appeared 4 months ago and the onset of menarche occurred 2 months ago. When she came to our clinic, she showed breast development (Tanner stage 3), the onset of the menstrual period and no pubic and armpit hair. She had a height of 138.2 cm (50th–97th) and weight of 45.9 kg (>97th), which based on 2009 height and weight standardized growth chart for Chinese children and adolescents aged 0 to 18 years old. Her body mass index (BMI) was 24.0 kg/m^2^ and the girl was considered obesity according to the 2004 overweight and obesity criteria for Chinese children and adolescents recommended by the Working Group for Obesity in China (WGOC).^[10]^ She presented with accelerated growth velocity (10 cm/yr) and the advanced bone age (11 yrs) (Table 1).

**Table 1:**
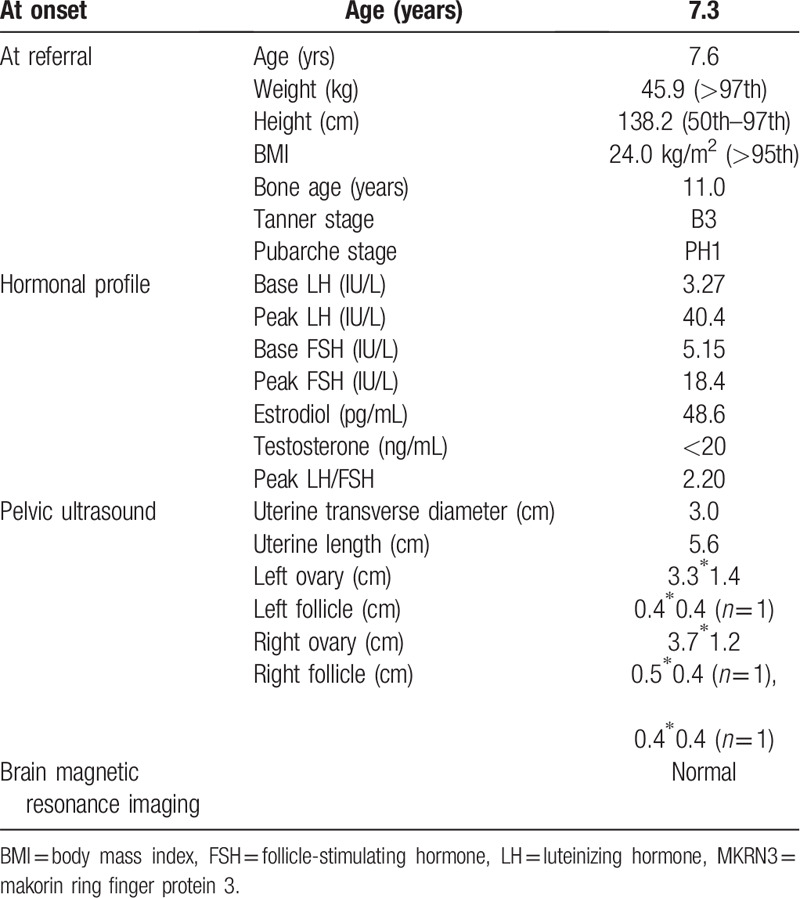
Clinical and laboratory features of the profound with the *MKRN3* mutation.

The patient was the second child born at full term with a birth weight of 4000 g and a birth height of 50 cm. Her parents were no consanguineous marriage. The height of her mother was 155 cm, and the age of menarche was 12 years. The height of her father was 170 cm, and the age of the first spermatorrhea was 12-13 years. The patient's sister is 16 years old now, and the age of menarche was 13 years. The paternal grandfather's height was 185 cm, and the age of puberty onset was unknown. The paternal grandmother's height was 170 cm, the age of menarche was unknown and the age of menopause was 46 years. Except for a height of 118.0 cm when she was 4 years old (50th–97th), nothing special was found in her personal and past histories.

Laboratory tests showed markedly elevated basal luteinizing hormone (LH) levels. The gonadotropin-releasing hormone (GnRH) stimulation test showed extremely elevated peak LH values and peak LH/follicle-stimulating hormone (FSH) values (Table 1). Basal LH values >0.6 IU/L, especially peak LH values >5 IU/L and peak LH/FSH values >0.6 have been used to support the diagnose of CPP.^[2]^ Pelvic B ultrasound showed the presence of ovarian follicles with diameters ≥0.4 cm. Uterine length also indicated the onset of puberty. Contrast-enhanced magnetic resonance imaging (MRI) did not disclose any abnormality in the pituitary (Table 1). The patient was also accompanied by impaired glucose tolerance (IGT) due to the 2-hour blood glucose during an oral glucose tolerance test (75 g glucose load) was 8.27mmol/L.

The girl was finally diagnosed with idiopathic CPP and treated with a GnRH analog (3.75 mg every 4 wks). During this treatment, gonadotropin and estradiol levels were decreased to a prepubertal level. The breast development was stopped and the advanced bone age remained unchanged. Until the manuscript was prepared, the girl has been treated with a GnRH analog for 1 year and 5 months. Basal LH was 0.13 IU/L and estradiol was 29.2 pg/mL. The length of the uterine was 5.1 cm. No ovarian follicle with the diameter ≥ 0.4 cm was found. The pubertal signs of the girl have since not progressed, which indicates that the GnRH analogs treatment is effective. Additionally, her BMI has slightly decreased in comparison with that before the GnRH analogs treatment (22.2 kg/m^2^ vs 24.0 kg/m^2^) though the girl was still considered to be obesity.

Genomic DNA of the patient and the parents was extracted from peripheral venous blood by using QIAamp DNA Blood Mini Kit (Qiagen, Germany). Exonic sequences were enriched by using Agilent's SureSelect Human All Exon 50Mb Kit (Agilent Technologies, Santa Clara, CA, USA). The enriched library was sequenced using the HiSeq2000 (Illumina, San Diego, CA, USA) with 90-bp paired-end runs. We then aligned the resulting reads to the human genome version 19 reference genome using the Burrows-Wheeler Aligner software (http://bio-bwa.sourceforge.net/). Single nucleotide polymorphism and indel discovery were performed with the Genome Analysis Toolkit (http://www.broadinstitute.org/gatk). Finally, the identification of variants in the coding region of *MKRN3* was confirmed with the use of polymerase-chain-reaction (PCR) amplification followed by sequencing of the products using the conventional Sanger method (Genetic analyzer 3130, Applied Biosystems, Foster City, CA, USA).

**Figure 1:**
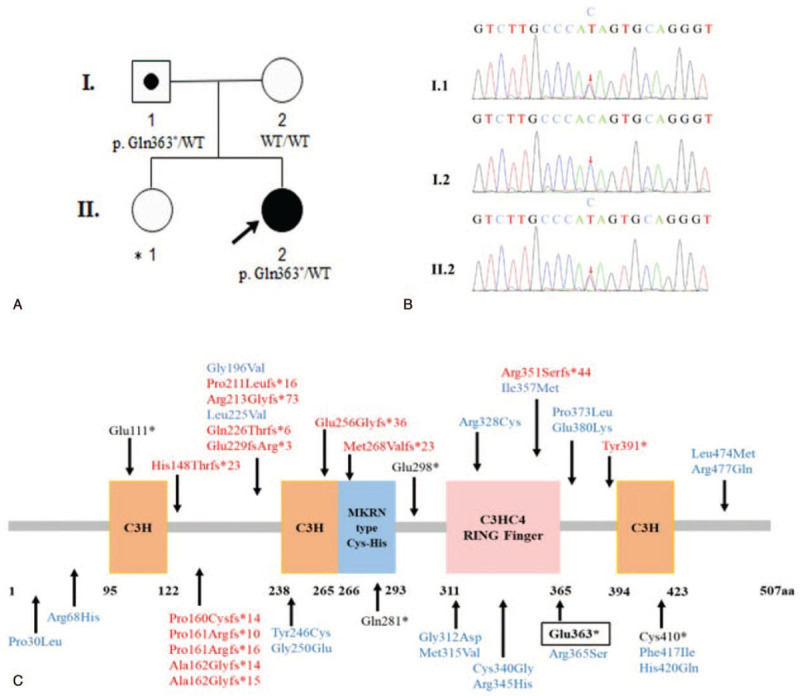
(A) Pedigrees of the investigated case with *MKRN3* mutation. Square indicates male family member, circles indicate female members, the black symbol indicates clinically affected family member, the symbol with black circle indicates unaffected carrier, ^∗^ indicates unknown genotype, the arrow indicates the profound in this family, WT, wild type. (B) Partial sequencing chromatographs of the *MKRN3* gene showing the novel heterozygous mutation (c. 1087C>T) detected in the profound. The same mutation was covered in her father but uncovered in her mother. (C) MKRN3 domains (three C3H zinc finger motifs, one C3H4 RING zinc finger motif, and one MKRN3 specific Cys-His domain) and *MKRN3* mutations identified in patients with CPP. The numbers correspond to the amino acid positions in the protein. Fourteen frameshift mutations (red), 15 missense mutations (blue), and 5 nonsense mutations (black) are shown, the black box indicates the nonsense mutation identified in our present study.

We found a novel heterozygous mutation consisting of base replacement (c.1087C>T). It was a nonsense mutation, which resulted in a premature stop codon that leading to a truncated protein (p. Gln363^∗^). This mutation was absent in the dbSNP137, the Exome Aggregation Consortium (ExAC), the genome aggregation database (gnomAD), and the 1000 Genomes Projects. In silico bioinformatic tools predicted the mutation to be pathogenic (Mutation Taster: 1, CADD: 37, DANN: 0.996, fathmm-MKL: 0.856). The girl's father carried the same heterozygous *MKRN3* mutation as the patient, and *MKRN3* mutation was absent from the girl's mother (Fig. 1A, B). Our present study was approved by the Ethics Committee of Beijing Children's Hospital, Capital Medical University, Beijing, China, and written informed consent was obtained from the patient and her parents.

## 3 Discussion

In this study, we identified the first Chinese *MKRN3* nonsense mutation causing CPP in a 7-year-old girl, who was subsequently effectively treated with GnRH analogs to control her pubertal development. This variant was inherited from her father who was an asymptomatic carrier of the same mutation.

Mutations in *MKRN3* have been identified as the most common genetic cause of CPP. It is estimated that in Western countries approximately 9% to 46% of familial CPP cases and 3% to 20% of sporadic CPP cases have identified *MKRN3* mutations.^[5]^ In 2013, *MKRN3* mutations were first identified as one of the causative factors of CPP in 5 of 15 families.^[7]^ Currently, 37 different loss-of-function mutations of *MKRN3* gene have been published in 86 cases with CPP, including 14 frameshift defects, 19 missense mutations, and 4 nonsense mutations (Table 2). However, the association between genotypes and clinical phenotypes has not been extensively explored. It is worth noting that the frequency of *MKRN3* mutations varies in different parts of the world, with more common in Western countries and relatively rare in Asian countries.^[5,8]^ Up to now, only 9 out of 37 *MKRN3* mutations, including one frameshift defect, seven missense mutations, and one nonsense mutation were reported in Asian countries (Table 2). However, the underlying mechanisms of the ethnic difference in the frequency of *MKRN3* mutations remain to be explored in the future. In addition, to date, all cases inherited mutations from their fathers, which are in accordance with the fact that *MKRN3* is a maternal imprinting gene. Our present study identified the first Chinese *MKRN3* nonsense mutation causing CPP in a 7-year-old girl, who inherited the mutation from her father.

**Table 2:**
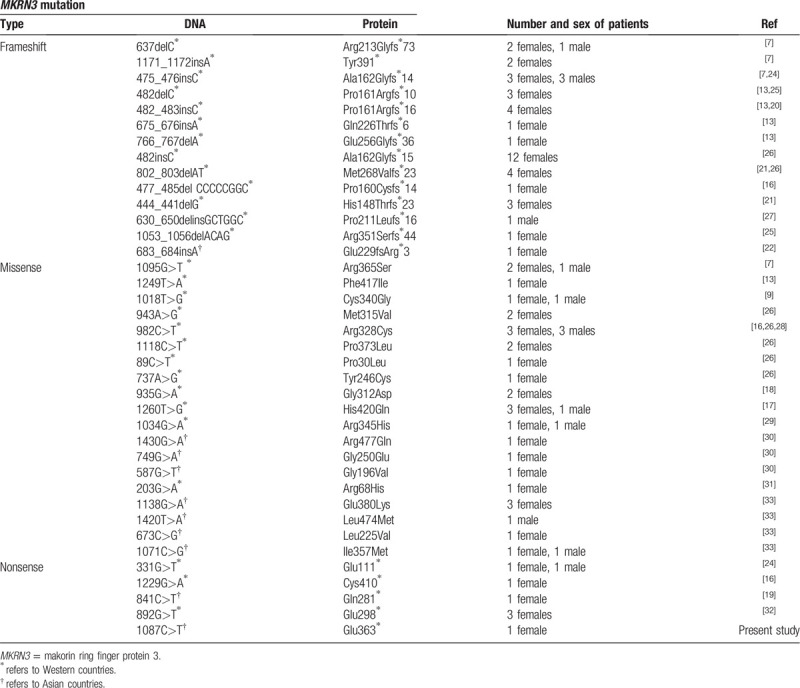
Epidemiological features of reported mutations in the *MKRN3* gene.

Although the causative effects of *MKRN3* mutations in CPP have succeeded in receiving the attention, in fact, the precise mechanisms of this gene triggering the onset of CPP are still unclear. *MKRN3* gene encoding the makorin ring finger protein 3, which includes 5 zinc-finger domains, 2 copies of a C3H motif in the N-terminal, then followed by a Cys-His configuration, a C3HC4 RING zinc-finger, and a final C3H motif as shown in Figure 1C.^[7]^ C3H zinc-finger motifs have been implicated in RNA binding, whereas the RING zinc-finger motif is involved in the ubiquitin-ligase activity.^[7]^ MKRN3 is associated with protein ubiquitination and has been postulated to serve as an inhibitor of GnRH secretion. Previously, Abreu et al^[7]^ studied in mice hypothalamic arcuate nucleus reported that *Mkrn3* mRNA levels were high in the prepubertal period, but decreased immediately before puberty and through puberty. Consistent with the results of animal studies, Hagen et al^[11]^ performed a population-based longitudinal study of 38 healthy Danish girls found that serum MKRN3 levels declined preceding pubertal onset and through puberty. Additionally, they also found the negative correlation of serum MKRN3 levels with FSH (*r* = -0.262, *P* = 0.015) and LH (*r* = -0.226, *P* = 0.037) in prepubertal girls, which further supported the major role of MKRN3 in inhibiting hypothalamic GnRH secretion during childhood.^[11]^ What's more, both Hagen et al^[11]^ and Lu et al^[12]^ demonstrated that serum MKRN3 levels were significantly decreased in CPP patients in comparison with that in the non-CPP controls. In this study, we identified a never reported novel heterozygosis nonsense mutation, which is in the C3HC4 RING zinc-finger, leading to a premature stop codon and resulting in a truncated protein (Glu363^∗^). We speculate that the protein expression terminated prematurely caused the decrease MKRN3 expression or the disappear enzyme activity, which further resulted in premature activation of GnRH secretion and, consequently, lead to the CPP development.

Previously, Macedo et al^[13]^ studied in 215 Brazil CPP patients demonstrated that compared with CPP patients without *MKRN3* mutations, CPP patients with *MKRN3* mutations had classical features of CPP and significantly higher basal FSH levels [4.9 (4.4–10) vs 3.6 (1.0–9.8), *P* < 0.05]. In consistent with their results, the girl with *MKRN3* mutation in our present study presented with typical rapidly progressive clinical features of CPP, including the menstrual onset after the breast development only 2 months, the advanced bone age, and the accelerated growth velocity. Hormonal profiles showed that the basal FSH levels were reasonably high to LH (5.15 IU/L vs 3.27 IU/L). It is noteworthy that the peak levels of both stimulated LH and FSH were significantly high in our female patient with *MKRN3* nonsense mutation (40.4 IU/L and 18.4 IU/L, respectively), which suggested the predisposition to developing premature ovarian failure (POF) later in life.^[14]^ Our recently published studies pointed out that CPP may serve as a prelude to hypogonadism.^[15]^ In all published papers on *MKRN3* till now, only Grandone et al^[16]^ reported a CPP case harboring the *MKRN3* mutation (Pro160Cysfs^∗^14) presented with the POF (premature menopause at 36 yrs). Therefore, a long-term clinical follow-up is necessary for our present case to evaluate the possible association between *MKRN3* mutations and POF.

Currently, long-acting GnRH analogs are the gold standard treatment for CPP.^[2]^ Previous reports show that CPP patients with *MKRN3* mutations seemed to have satisfactorily controlled pubertal development after being treated with GnRH analogs although information regarding the dose of the medicine and response to treatment was only observed in almost one-third of the cases (24 out of 79 cases).^[13,16–22]^ In our present female patient, after 1 year and 5 months treatment with GnRH analog (3.75 mg every 4 wks), gonadotropin and estradiol levels were decreased to prepubertal level and the pubertal signs of the girl have since not progressed, which indicate that the GnRH analogs treatment is effective.

However, it is worth mentioning that GnRH analogs treatment has been reported to be associated with a high risk of polycystic ovary syndrome (PCOS).^[23]^ Our present case was obese accompanied by the IGT, which could also contribute to the development of PCOS. Furthermore, both early puberty and obesity have been documented to be associated with adverse long-term endocrine and metabolic outcomes, including hypertension, type 2 diabetes, stroke, cardiovascular mortality, and cancer. Thus, we emphasized the importance of weight control and clinical follow-up. At present, after following up 1 year and 5 months, her BMI has slightly decreased (22.2 kg/m^2^ vs 24.0 kg/m^2^) though the girl was still considered to be obesity. No other endocrine disorders were found. Given our present study is the first to report *MKRN3* nonsense mutation in a CPP girl, who also has obese companied with IGT, weight management and the long-term follow-up was still needed in order to monitor the adverse health outcomes, especially for the PCOS in later life.

In conclusion, *MKRN3* mutations represent the most frequent genetic cause of CPP. Here, we present a novel heterozygous *MKRN3* nonsense mutation in a 7-year-old Chinese rapidly progressive CPP girl, supporting the role of *MKRN3* in the control of puberty development. Additionally, our present case was obese companied with IGT at the baseline assessment and exhibited stronger LH/FSH response to GnRH stimulation test. Compare with the literature reported, we address that the ovary function should be paid more attention and weight management and the long-term follow-up was important in order to monitor the adverse health outcomes, especially for the PCOS in later life.

## Acknowledgments

The authors would like to thank the family members of the patients involved in this study.

## Author contributions

Meijuan Liu reviewed the literature and wrote the primary manuscript. Lijun Fan collected the clinical data and completed the genetic analysis. Chunxiu Gong diagnosed the patient, provided follow-up, and revised the primary manuscript. All of the authors read and approved the final manuscript.

**Data curation:** Lijun Fan.

**Funding acquisition:** Chun Xiu Gong.

**Supervision:** Chun Xiu Gong.

**Writing – original draft:** Meijuan Liu.

**Writing – review & editing:** Chun Xiu Gong.

## Abbreviations

Abbreviations: BMI = body mass index, CPP = central precocious puberty, DLK1 = delta-like non-canonical Notch ligand 1, ExAC = exome aggregation consortium, FSH = follicle-stimulating hormone, gnomAD = genome aggregation database, GnRH = gonadotropin-releasing hormone, IGT = impaired glucose tolerance, KISS1 = kisspetin 1, KISS1R = kisspetin 1 receptor, LH = luteinizing hormone, MKRN3 = makorin ring finger protein 3, MRI = magnetic resonance imaging, NR0B1 = nuclear receptor subfamily 0 group B member 1, PCOS = polycystic ovary syndrome, PCR = polymerase-chain-reaction, POF = premature ovarian failure, PROKR2 = prokineticin receptor 2, PWS = prader-willi syndrome, WGOC = working group for obesity in China.
